# Oxidative stress on vessels at the maternal-fetal interface for female reproductive system disorders: Update

**DOI:** 10.3389/fendo.2023.1118121

**Published:** 2023-03-10

**Authors:** Chenlu Zhang, Yaxin Guo, Yan Yang, Zhaojin Du, Yunhui Fan, Yin Zhao, Suzhen Yuan

**Affiliations:** ^1^ Reproductive Medicine Center, Tongji Hospital, Tongji Medical College, Huazhong University of Science and Technology, Wuhan, Hubei, China; ^2^ School of Optical and Electronic Information, Huazhong University of Science and Technology, Wuhan, China; ^3^ Reproductive Medical Center, Qingdao Women and Children's Hospital, Qingdao University, Qingdao, China; ^4^ Department of Orthopedics, Tongji Hospital, Tongji Medical College, Huazhong University of Science and Technology, Wuhan, Hubei, China; ^5^ Department of Ophthalmology, Tongji Hospital, Tongji Medical College, Huazhong University of Science and Technology, Wuhan, Hubei, China; ^6^ Department of Obstetrics and Gynecology, Tongji Hospital, Tongji Medical College, Huazhong University of Science and Technology, Wuhan, Hubei, China

**Keywords:** maternal-fetal interface, oxidative stress, reactive oxygen species, blood vessels, signaling mechanism

## Abstract

Considerable evidence shows that oxidative stress exists in the pathophysiological process of female reproductive system diseases. At present, there have been many studies on oxidative stress of placenta during pregnancy, especially for preeclampsia. However, studies that directly focus on the effects of oxidative stress on blood vessels at the maternal-fetal interface and their associated possible outcomes are still incomplete and ambiguous. To provide an option for early clinical prediction and therapeutic application of oxidative stress in female reproductive system diseases, this paper briefly describes the composition of the maternal-fetal interface and the molecular mediators produced by oxidative stress, focuses on the sources of oxidative stress and the signaling pathways of oxidative stress at the maternal-fetal interface, expounds the adverse consequences of oxidative stress on blood vessels, and deeply discusses the relationship between oxidative stress and some pregnancy complications and other female reproductive system diseases.

## 1 Introduction

At present, many studies have confirmed the correlation between oxidative stress and female reproductive system diseases. This direction has become one of the hot spots in current clinical and basic research. Oxidative stress during pregnancy is associated with many adverse pregnancy outcomes. Despite these recognized associations, the underlying mechanism is not fully understood. A great deal of work has been done on preeclampsia, but in order to better understand the mechanism of oxidative stress on placental blood vessels, we need to broaden the range of complications of pregnancy beyond PE, such as intrauterine fetal growth restriction, miscarriage, premature birth with low birth weight, etc. This review systematically introduces the components of the maternal-fetal interface and the molecular mediators closely related to oxidative stress, and details the possible sources of oxidative stress at the maternal-fetal interface. Compared with previous work, it innovatively summarizes the contribution of external and psychological factors to the sources of placental oxidative stress. In addition, this paper focuses on the effects of oxidative stress on placental blood vessels, elaborates the signaling pathway of oxidative stress at the maternal-fetal interface, and describes the adverse consequences of oxidative stress on blood vessels in three aspects: abnormal development of placental blood vessels, immune disorders and endothelial cell dysfunction. Futhermore, the internal relationship between oxidative stress and some pregnancy complications as well as other female reproductive system diseases was discussed from the perspective of blood vessels. Finally, further research spaces and prospects are proposed in order to provide more options and treatment ideas for early clinical prediction of oxidative stress in female reproductive system diseases.

### 1.1 Maternal-fetal interface

The maternal-fetal interface is a direct contact between maternal tissue and fetal components. The placenta is an important interface between the mother and fetus (1, 2). The placenta is composed of fetal amniotic membrane, chorion frondosum as well as decidua basalis from differentiated maternal endometrial stromal fibroblasts. The chorion contains branches of the umbilical cord, from which many villi of different sizes emanate. After implantation of the late blastocyst, as trophoblast cells invade the uterine wall, the uterine spiral artery will rupture and open directly into the intervillous space, which will therefore be filled with maternal blood. These free villi are suspended in the maternal blood, absorbing oxygen and nutrients as well as excreting metabolites. In addition, uterine spiral artery remodeling, which is closely related to extravvillous trophoblast (EVT) invasion, is also a key factor in the formation of maternal-fetal interface (3). As a result, the placenta is also a vascular organ. The placenta is capable of exchanging gases, nutrients, metabolites, and produces a variety of hormones and growth factors that support normal fetal development and maintain a healthy pregnancy (4). The placenta is a vital organ for maintaining the growth and development of the fetus (5).

In physiological conditions, maternal blood and fetal blood are not directly connected, separated by the villous capillary wall, villous interstitium, and villous cytotrophoblasts, which constitute the maternal-fetal interface and play the role of material exchange and placental barrier. The placental villi at this time are tertiary villi composed of fetal capillary network, mesenchyme, cytotrophoblast, and syncytial trophoblasts (2). These four elements constitute the placental barrier, jointly play a part in maternal-fetal interface (6). The placental barrier is the barrier between the placental villi and the uterine blood sinusoids, which has limited defense function. Placenta also has an immune protection function, and its local immune response plays an important role in the establishment and maintenance of pregnancy and the initiation of labor. As a kind of semi-allograft, the fetus can be tolerated by the uterus, which maybe related to the following possible reasons: the balance and transformation between maternal immune rejection and immune tolerance to the fetus, the generation and outcome of a large number of inflammatory factors and immune cells (7), as well as the absence of antigenicity of early embryonic tissue.

**Figure 1:**
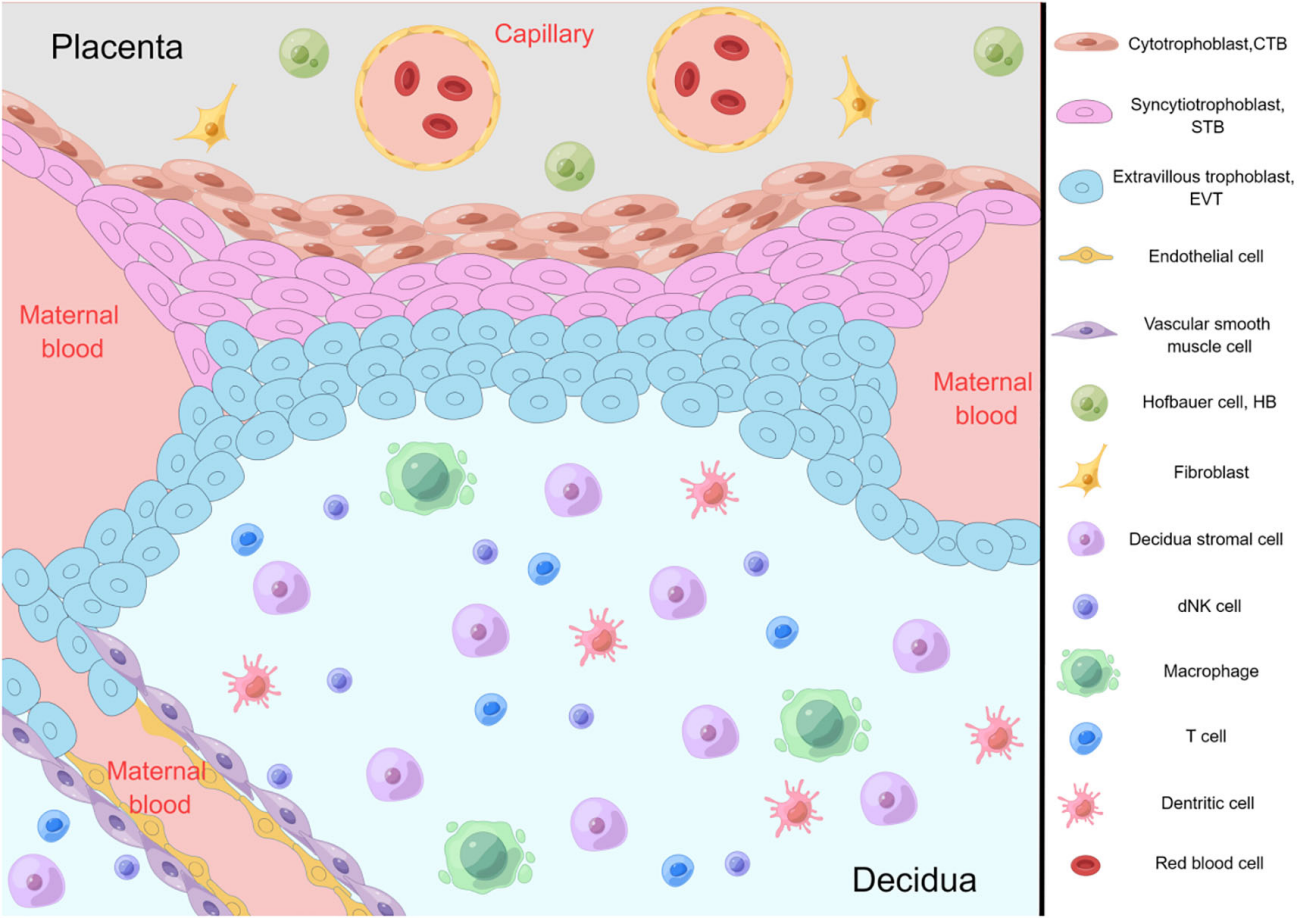
Diagram of cellular components at the maternal-fetal interface. By Figdraw.

In order to explore the classification of cell subtypes at the maternal-fetal interface shown in **Figure 1**, many researchers have conducted single-cell sequencing and single-cell transcriptome studies on human placentas which are at different gestational stages. By analyzing single-cell RNA sequences from human placental villus and decidua tissues at 11-13 weeks of gestation, Sun et al. identified five major cell types (trophoblasts, stromal cells, Hofbauer cells, antigen presenting cells, and endothelial cells) and unique interactions among them (8). At the same time, they found that the sex of the fetus affects pregnancy, because some chemokine genes were found to be differentially expressed in trophoblast cells and Hofbauer cells, indicating that placental immune function is sexually dimorphic. This is also a strong argument for the sex-biased expression of specific genes found in previous studies of human placentas in early and full-term gestation (9–11). Liu et al. conducted cell sorting of placentas from 8 and 24 weeks of gestation and identified 14 different subtypes of placental cells by single-cell transcriptome profiling (12). Three subtypes of CTB cells (CTB_8W_1, CTB_8W_2, and CTB_8W_3) found in the first trimester of pregnancy have different degrees of division and differentiation potential, which may be the origin of CTB and STB, respectively. As the placenta grows and adapts to maternal changes during normal pregnancy, its cell ratio and gene expression change dynamically. In this retrospective review of human placental scRNA-seq by Li et al., large transcriptional differences between placentas at different sites and gestational ages were also demonstrated (2). Elucidating cell subtypes at the maternal-fetal interface will help to understand the cellular pathways and mechanisms that may exist in the physiological and pathological conditions of pregnancy, and provide a way to discover the underlying causes of adverse pregnancy outcomes.

### 1.2 Oxidative stress

As an essential element in nature, oxygen is also essential for life activities. Oxidative metabolism is a significant source of energy for life. The latest concept of oxidative stress is that “An imbalance between oxidants and antioxidants in favor of oxidants, leading to a disruption of redox signaling and control and/or molecular damage” (13). And there are many different specific forms of oxidative stress, including: Nutritional oxidative stress, Dietary oxidative stress, Postprandial oxidative stress, Physiological oxidative stress, Photooxidative stress,{Ultraviolet (UV-A, UV-B), Infrared-A}, Radiation-induced oxidative stress, Nitrosative stress, Reductive stress.

As the main molecular switch of oxidative stress (14), reactive oxygen species (ROS) are mostly generated by electron leakage from the electron transport chain (ETC) during mitochondrial oxidative metabolism (15). The most important ROS in organisms include superoxide anion (O_2_−•), hydroxyl radical (•OH), peroxygen (ROO•), alkoxy (RO•) and hydroxyl peroxygen (HO_2_•) (16), among which there are mainly three types: superoxide anion (O_2_−•), hydrogen peroxide (H_2_O_2_) and hydroxyl (•OH). These radicals are unstable and can react with various components of living organisms, such as lipids, nucleic acids, proteins, carbohydrates, or nearby molecules. However, different ROS have different selectivity for target molecules (14). For example, hydroxyl radicals (HO•) are indiscriminately reactive to biomolecules, whereas O_2_ - and H_2_O_2_ each have preferred biological targets. Superoxide anion (O_2_−) can act on active iron-sulfur (Fe-S) clusters; However, hydrogen peroxide (H_2_O_2_) is active on Cys residues. There are two kinds of antioxidants in living organisms: enzymatic and non-enzymatic. Enzymatic antioxidants include superoxide dismutase(SOD), blood oxygenase(HO-1), catalase(CAT), glutathione peroxidase (GPx) and thioredoxin (TRX). Vitamin C, vitamin E, glutathione, taurine, hypotaurine, zinc, selenium (Se), β-carotene, carotene, nicotinamide adenine dinucleotide (NADH), and nicotinamide adenine dinucleotide phosphate (NADPH) are all non-enzymatic antioxidants.

In the study of molecular signaling mechanisms, the discovery of phosphorylation/dephosphorylation is the beginning of the exploration in the field of molecular switches (17). In the molecular switch of redox, especially in the study of redox proteome, the dynamic change and effect of cysteine are the most momentous (18). The link between phosphorylation/dephosphorylation and protein cysteine reduction/oxidation is related to the REDOX sensitivity of key cysteine residues in protein phosphatases. This elucidates the molecular pathway of the signaling cascade, which is the basic process of various signal transduction pathways in biology (19). Reactive oxygen species (ROS), as second messengers in many life processes, make a difference in intracellular signaling cascades (20). Reactive oxygen species are key signal components for cell growth and tissue development (21). Normal levels of ROS play a vital regulatory role in follicular genesis, luteal growth, oocyte maturation, and development of fetal placental through a variety of signal transduction pathways (22). To maintain the homeostasis between cells and the surrounding environment, ROS exists subcellular colocalization and its target in complex mammals, which makes ROS signaling specific and plays a role in various signaling pathways. Examples include programs regulated by tumor suppressor p53, peroxisome proliferator-activated receptor-γ (PPARγ) coactivator 1α, oncogene C-MYc, and class O forkhead box transcription factors (FOXOs). The KEAP1-NrF2 pathway can also regulate oxidative stress and stress protective response, which involves a cyanozinc redox center (23). In addition, reactive oxygen intermediates also function as apparently widely used messengers in the NF-Kappa B pathway (24). These pathways may provide a long-lasting oxidative protective response or cause cell death to remove cells damaged by oxidation.

**Figure 2:**
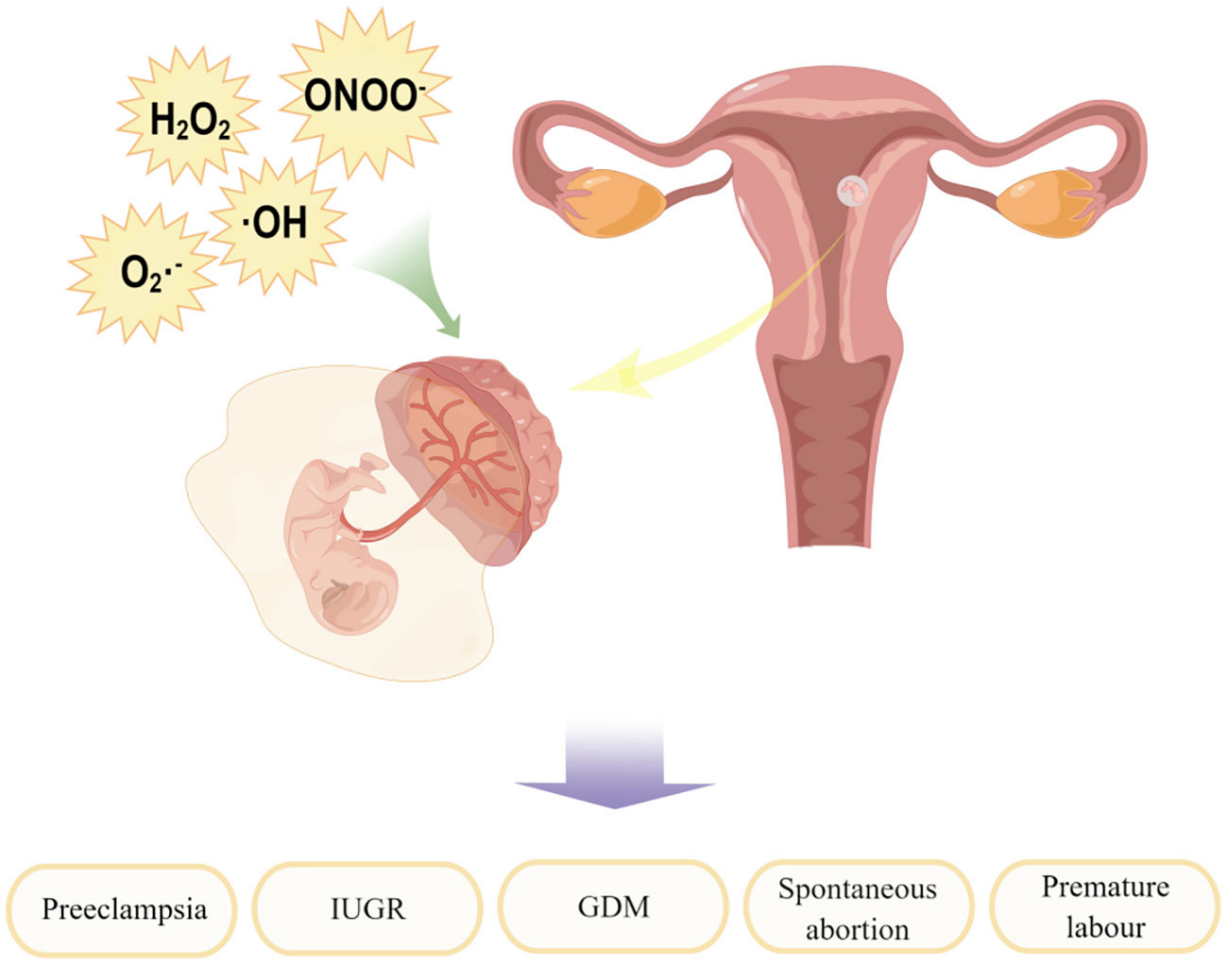
Factors contributing to the development of oxidative stress and their impact on maternal-fetal interface. By Figdraw.

Sies (19) divided oxidative stress into three levels: physiological oxidative stress, excess, and toxic oxidative load. In other words, in normal physiological conditions, oxidative stress also exists in the human body, which is essential for many metabolic processes and cell survival (25). However, it can also be a double-edged sword: too much ROS can have a damaging effect when the production of reactive oxygen species overpowers the intrinsic antioxidant defense system. The outermost shell of a free radical atom contains one or more unpaired electrons, making the radical highly reactive. When free radicals acquire electrons from biomolecules, they become stable, causing a cascade of chain reactions that lead to cell damage, such as DNA damage, lipid peroxidation, and protein damage (26–28), which may then lead to various female reproductive system diseases (29, 30), such as embryo reabsorption, recurrent pregnancy loss, preeclampsia, intrauterine growth restriction (IUGR) and fetal death (31). A full treatment of the problem awaits further treatment (in preparation). Further molecular understanding will also deepen the impact on translational medicine. It is important to study the molecular mediators of oxidative stress, which will provide possible therapeutic targets for female reproductive system diseases, as shown in **Figure 2**.

## 2 Sources of oxidative stress at the maternal-fetal interface

### 2.1 Excess production of oxidants

**Figure 3:**
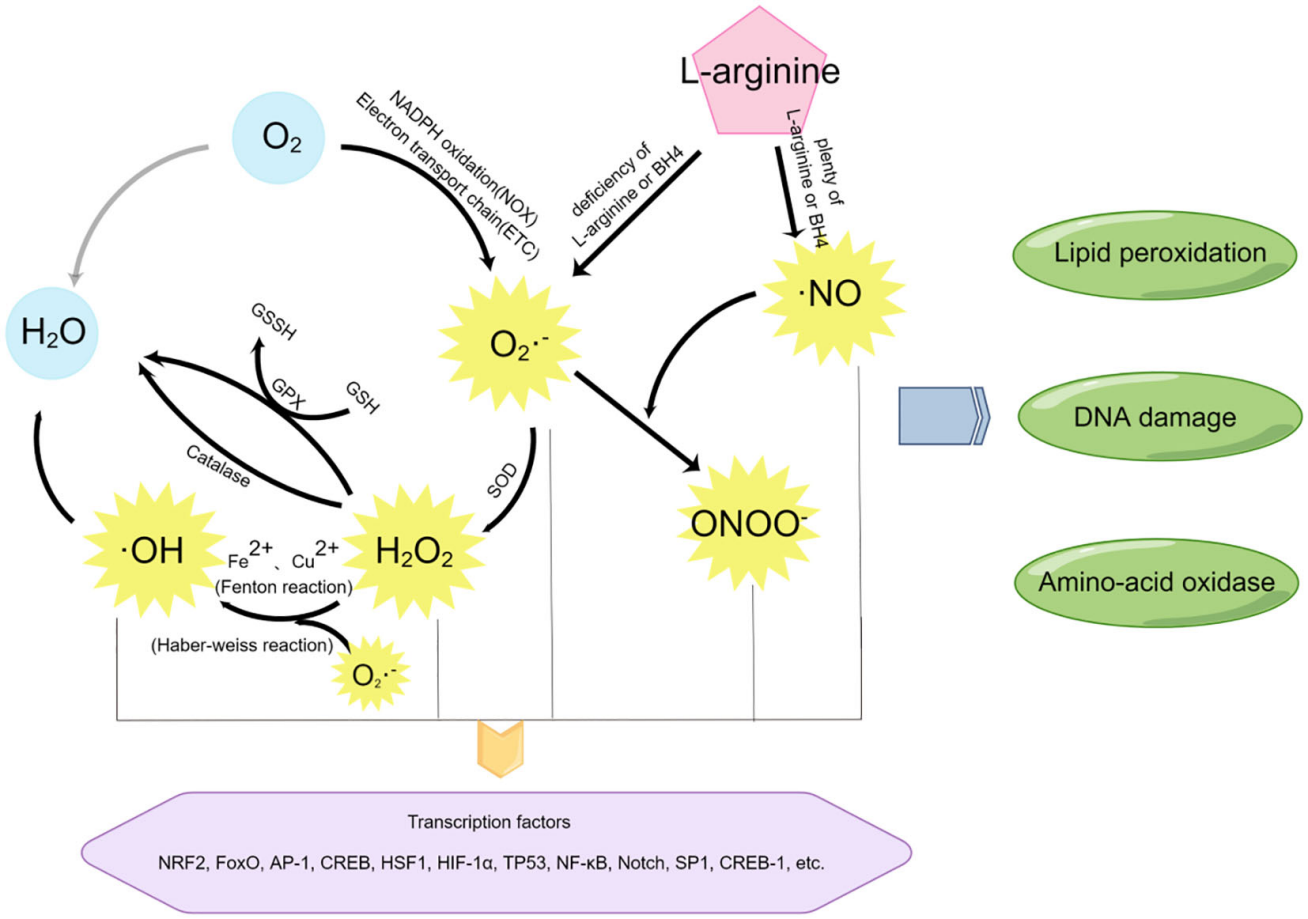
The mediators of oxidative stress and nitrification stress and their related reaction pathways. By Figdraw.

An equally important parallel process to oxidative stress at the maternal-fetal interface is nitrification stress. The molecular switches of nitrification stress are reactive nitrogen species (RNS), mainly including nitric oxide (•NO) and peroxynitrite (ONOO−). In the metabolism of aerobic organisms, it is quite normal for the production of ROS and RNS, which function as second messengers in many signaling cascades (32). Due to their high reactivity, these free radicals also have potential biological toxicity (33). Excessive generation of free radicals and other oxidizing substances may destroy the natural antioxidant defense system of the human body and create an environment unsuitable for normal female physiological reactions (34), at which time OS and NS will occur. Several different metabolic pathways may produce ROS and RNS, as shown in **Figure 3**.

Most free radicals are produced by the O_2_ itself. Using O_2_ as the reaction substrate, a large amount of ROS is generated through the oxidation reaction of the mitochondrial respiratory chain (also known as electron transport chain (ETC)) or nicotinamide adenine dinucleotide phosphate (NADPH), among which superoxide (SO) anion (O_2_−•) is the most common (35). In addition, superoxide (SO) anion (O_2_−•) can also be generated through short electron chains of the endoplasmic reticulum (ER), cytochrome P450, and other oxidoreductases (20, 36). These pathways are essential for ROS production at the maternal-fetal interface during early pregnancy.

Superoxide (SO) anion (O2−•) can be converted to H_2_O_2_ by superoxide dismutase (SOD). On the one hand, the production of H_2_O_2_ will activate the biological protection system through catalase metabolism or glutathione peroxidase (GPx) catalysis, H_2_O_2_ will be oxidized and metabolized into H_2_O and O_2_; On the other hand, H_2_O_2_ can act as an intermediate of the more toxic hydroxyl radical (•OH) and undergo subsequent reactions.

Highly active hydroxyl radical (•OH) is mainly generated by Haber-Weiss reaction (37) and Fenton reaction (33). In the Haber-Weiss reaction, superoxide (SO) anion (O_2_−•) reacts with H_2_O_2_ to generate more toxic free radicals detrimental to biological systems. Studies have shown that DNA strands are prone to break after the modification of bases by hydroxyl radicals (•OH), leading to DNA damage (38).

RNS include nitric oxide (•NO), which is formed by L-arginine in response to NO synthase (eNOS). At low concentrations of the substrate l-arginine or the cofactor BH4, superoxide (SO) anion (O_2_−•) and H_2_O_2_ are produced. However, nitric oxide (•NO) can further react with superoxide (SO) anion (O_2_−•) to generate peroxynitrite (ONOO−) (20), thus causing nitrification stress (NS) (39).

### 2.2 Deficiency of antioxidant

Antioxidants act as cleaners, removing excess ROS and RNS from our body. Without antioxidants in the body, the relationship between oxidation and antioxidation will be out of balance. Lack of antioxidants is also an important cause of oxidative stress (40), which has a devastating impact on human reproduction.

There are two types of antioxidants in the body, one is enzymatic and the other is non-enzymatic. Superoxide dismutase (SOD) (36), blood oxygenase(HO-1), catalase(CAT), glutathione peroxidase(GPx) (41) and thioredoxin (TRX) are enzymatic antioxidants. Their common feature is that they all contain a metal center, which is a key factor to their detoxification by transferring electrons to neutralize excess free radicals and balance different molecules, thus protecting the cell from structural damage. Another essential type of antioxidant is non-enzymatic, including vitamin C, glutathione (42), taurine, hypotaurine (43), vitamin E(α-tocopherol), zinc, selenium(Se), β-carotene, carotene, nicotinamide adenine dinucleotide (NADH) and nicotinamide adenine dinucleotide phosphate (NADPH), among which vitamin E is widely present in all cell membranes. It can prevent lipid peroxidation. The iron-binding proteins transferrin and ferritin can exert antioxidant effects through chelation (44). Furthermore, melatonin is an endogenous, non-enzymatic antioxidant secreted by the pineal gland (45, 46). It can react with various free radicals to form related metabolites; for example, melatonin can react with two ·OH to form Cyclic 3-hydroxymelatonin (C3OHM) (47–49). But melatonin is not as reusable as other antioxidants, such as glutathione, through the REDOX cycle.

### 2.3 Dysfunction of mitochondria

The eukaryotic mitochondrial network is perhaps the most complex and dynamically responsive sensing system. It integrates metabolism, oxygen, and danger signals from other organelles. Mitochondria have a profound influence on human health and disease. In addition to producing ATP through oxidative metabolism, mitochondria can also respond to external harmful signals by inducing inflammatory responses. In particular, mitochondrial reactive oxygen species is a key signal to start inflammatory responses, thus causing oxidative stress.

Oxidative stress damage caused by ROS in pathological conditions is a common feature of many diseases, and the sources of ROS in cells are extensive. Since reactive oxygen species from mitochondria account for the majority of the whole reactive oxygen species, oxidative metabolism of mitochondrial is the most important way to generate ROS (15), so mitochondria are also vulnerable to damage caused by reactive oxygen species, leading to their abnormal function. An appropriate amount of ROS can stimulate cell division and growth, but any problem occurring in mitochondria will affect the energy metabolism process, resulting in excessive ROS. These excessive ROS will in turn further damage the function of mitochondria of embryonic cells and oocytes. Superoxide anions in the mitochondrial matrix have high reactivity, which can damage mitochondrial DNA and proteins, and change mitochondrial genes (50). Mitochondrial DNA contains many genes encoding essential enzymes in the mitochondrial respiratory chain. Oxidative damage to DNA will inhibit the expression of key proteins in the respiratory chain, thereby increasing the content of ROS and damaging the regulatory function of mitochondria, thereby causing mitochondrial dysfunction (51). In addition, mitochondria are organelles composed of internal and external membranes. Excessive ROS oxidizes the phospholipid bilayer, changes the permeability of the mitochondrial membrane, and leads to negative changes in many mitochondrial functions (52, 53). These factors further stimulate an environment of oxidative stress, creating a self-reinforcing cycle.

If excessive mitochondrial reactive oxygen species, especially superoxide anions, are produced beyond the scavenging capacity of peroxide dismutase, excessive H_2_O_2_ will react with ferrous ions and copper ions to produce hydroxyl radicals (•OH) with higher activity (54), which will damage cell functions and cause damage to DNA, proteins and lipids. This is associated with the onset of many diseases, such as hypertension and insulin resistance.

### 2.4 Produced by placental cells

Through single cell sequencing of the placenta, it was found that stromal cells, endothelial cells, villous cytotrophoblast cells (CTV) and extra-villous trophoblast cells (EVT) are relatively abundant in placental cells (55). It has previously been shown that in various highly vascularized tissues, such as the brain, oxidative stress states can be induced by hypoxia, hyperoxia, or hypoxia-reoxygenation alternans (56). The characteristic hypoxic state in the placental microenvironment during early pregnancy makes the subsequent uterine spiral artery remodeling a process of “chronic intermittent hypoxic reoxygenation”, whose pathophysiology is similar to that of ischemia-reperfusion injury, which leads to the generation of reactive oxygen species (ROS). Oxidative stress may also be associated with non-trophoblast placental cells, such as endothelial cells (ECs), villous stromal cells, or immune cells (Hofbauer cells) in placental tissue. Sisino et al. (57) found that Hofbauer cells may induce the production of nitric oxide (•NO) in certain circumstances and produce peroxynitrite (ONOO−) through oxidative stress, leading to nitrification stress. Trophoblast cells can release exosomes rich in inflammatory substances (58) into the maternal blood circulation, affecting maternal endothelial cells; Moreover, placenta also releases some cell debris containing apoptotic bodies (59) and some antiangiogenic factors (60, 61) into the maternal circulation, which may be involved in the development of oxidative stress. Placental hypoxia can stimulate the production of damage-associated molecular patterns (DAMP), activate immune cells such as neutrophils, produce proinflammatory cytokines such as tumor necrosis factor-α(TNFα), and promote oxidative stress by activating neutrophil nicotinamide adenine dinucleotide phosphate (NADPH) oxidase.

### 2.5 External stressors

The external factors causing oxidative stress include chemical stressors and physical stressors. Among them, chemical stressors are the main external factors, including exogenous oxidants (such as H_2_O_2_, cigarette smoke extract (CSE: OS inducer verified in fetal cells) (62)) and endogenous oxidants (such as neurotoxin 1-methyl-4-phenylpyrix). It also includes the improper use of some drugs that have toxic effects on the body (such as doxorubicin). In many oxidative stress models constructed *in vitro*, H_2_O_2_ is the most common chemical stressor, which is not only cheap and stable, but also has a strong oxidative stress effect (63, 64). In addition, flame retardants such as polybrominated diphenyl ethers (PBDE, such as BDE-47) can also cause oxidative stress in the body (65). DCVC (S-(1, 2-dichloroethylene) -L-cysteine), a metabolite of the chemical trichloroethylene (TCE), induces ROS generation at the maternal-fetal interface, which causes mitochondrial dysfunction, releases IL-6, and thus stimulates inflammation (66). Physical stressors cause oxidative damage to the body mainly through ultraviolet irradiation, irradiation, and hypoxia/reoxygenation (H/R) operation. For example, there have been many studies on HTR8/SVneo cell lines exposed to hypoxia/reoxygenation (H/R) conditions (67–69).

### 2.6 Mental and psychological factors

At present, studies have shown that psychosocial stress is related to the increase of biomarkers of oxidative damage in human serum (70, 71), which indicates that psychosocial factors are also an important source of oxidative stress. In a study of psychological stress and oxidative stress in pregnant women, psychosocial status and stressful life events (SLEs) were evaluated by two scales respectively. Levels of 8-isoprostaglandin F2_α_ (8-iso-PGF2_α_), a relatively stable biomarker of oxidative stress in serum, were also found to be higher in pregnant women with low socioeconomic status and high stress during pregnancy (72). However, the assessment of pressure in this work is limited, and whether there are other fields of pressure and measurement methods and the establishment of accurate measurement of perceived pressure is still worth exploring in the future.

## 3 Mechanisms of oxidative stress on blood vessels at maternal-fetal interface

Normal pregnancy is closely related to the development of placental villous blood vessels and the establishment of placental blood flow in low-resistance uterus, which includes the establishment of two pivotal links: fetoplacental circulation and utero-placental circulation. In normal human placenta, physiological hypoxia can be detected by *in vivo* methods during the first trimester of pregnancy (73). This may suggest that the presence of an anoxic environment provides the necessary signals for development (74). This idea may also be justified from an evolutionary point of view, since primitive life also began in near-anaerobic conditions and developed gradually in anoxic conditions. This hypoxic environment will be improved at the end of early pregnancy (12-14 weeks of pregnancy) due to the initiation of vascular remodeling process. Meanwhile, vascular endothelial cells are replaced by endovascular extravillous trophoblast cells (enEVT) subsets, which leads to vascular remodeling. This process can increase blood flow and the partial pressure of oxygen, which transfers placental blood vessels from “low discharge and high resistance” to “low resistance and high discharge”. At this time, the utero-placental circulation is established (75). This process is usually completed at 20 weeks of gestation (76).

Therefore, the placenta is a vascular organ at the maternal-fetal interface, and the formation of placental angiogenesis is related to all kinds of cells at the maternal-fetal interface, especially trophoblast cells, endothelial cells and endometrial cells (decidual cells). Among them, trophoblast cells play a crucial role in the change of oxygen partial pressure environment, because extravillous trophoblasts are the key to the remodeling of uterine spiral artery. When there is oxidative stress at the maternal-fetal interface, many pathological changes will be caused accordingly. Oxidative stress may change the partial pressure of oxygen at the maternal-fetal interface, exposing cells to an inappropriate oxygen environment too early or too late, thus causing different degrees of dysfunction of cells through different pathways. For example, oxidative stress damage to trophoblast cells may cause abnormal development of placental blood vessels, and the effects on endothelial cells may cause damage to blood vessels more directly. The possible pathways of oxidative stress on the cells that make up the placental blood vessels and the corresponding consequences are discussed below.

### 3.1 Signaling mechanisms of oxidative stress

The continuous production of excessive free radicals will lead to negative results in many signaling pathways of life activities (22), and even lead to apoptosis and necrosis of cells. In some pathways, free radicals participate in reactions as direct destructive agents, while in others they act as intermediate signaling molecules – second messengers (77). ROS can activate some redox-sensitive transcription factors, such as NRF2, FoxO, AP-1, CREB, HSF1, HIF-1α, TP53, NF-κB, Notch, SP1 and CREB-1 to regulate cytokine expression. Burdun et al. (20) described in detail the molecular signaling mechanism of adverse consequences caused by oxidative stress, including but not limited to affecting ion channels, leading to lipid peroxidation, DNA damage, and protein modification.

#### 3.1.1 Effects on ion channels

ROS affects the activity of phospholipase C (PLC), a key enzyme that controls calcium influx, and affects the activity of calcium ATPase (SERCA) in the endoplasmic reticulum (78). Calcium ions are released from the endoplasmic reticulum and the mitochondrial membrane potential is unstable, which increases the mitochondrial permeability and ultimately affects the functions of the mitochondrial electron transport chain related to ATP production. Moreover, calcium ion, as an important second messenger involved in many physiological processes, is also affected. ROS can also downregulate the expression of calcium-activated potassium channels KCa2.3 and KCa3.1 (KcaS), which are closely related to vasodilatation. This can enhance vascular contractility and increase blood pressure.

#### 3.1.2 lipid peroxidation

Oxidative stress can also damage cells through lipid peroxidation and destroy polyunsaturated fatty acids on cell membranes and/or organelle membranes through oxidation (79), thereby destroying membrane integrity and increasing membrane permeability (80). This affects the contents inside the membrane, for example, by inactivating enzymes and damaging DNA. Increased oxidative stress (81–83), and/or decreased activity of antioxidant enzymes, such as SOD, GPx, and TRX, leads to lipid peroxidation in the placenta (81), which may be an important reason for the formation of pathological placenta such as preeclampsia placenta. Especially in placenta of preeclampsia patients with hemolysis, elevated liver function and low platelet count syndrome(HELLP syndrome), carbonyl groups in proteins are significantly increased (84).

#### 3.1.3 DNA damage

Oxidative stress can induce nucleic acid damage. Many studies have confirmed this view. Oxidative stress can lead to DNA damage in the form of DNA strand break, DNA site mutation, DNA double strand aberration and mutation of proto-oncogenes and tumor suppressor genes. In an *in vivo* and *in vitro* cell study of DNA double-strand breaks labeled by γH2AX immunohistochemistry (85), it was found that the positive expression of γH2AX in the placenta of preeclampsia was significantly higher than that of normal controls, especially in maternal decidua cells, which suggested that there may be factors related to DNA damage in placenta of preeclampsia. For primary cultured trophoblast cells and decidual cells *in vitro*, no matter by giving 100uM hydrogen peroxide (H_2_O_2_) or creating an ischemia-reperfusion environment to achieve the purpose of artificial oxidative stress environment, the results showed that the DNA damage of decidual cells was more severe. It is known that 8-hydroxy-20-deoxyguanosine (8-OH-dG) can modify oxidized damaged bases, and detection of placental cell DNA in preeclampsia patients with IUGR can also find a higher concentration of labeled cells (86, 87).

#### 3.1.4 Protein posttranslational modification

Oxidative stress can process proteins through posttranslational modification, which changes the expression products from the perspective of epigenetics, thus affecting their activity and function. At present, the widely recognized mechanism is the transfer of nitrogen on protein tyrosine residues caused by nitrification stress, especially the protein modification by peroxynitrite (ONOO−), which may activate the expression of active protein molecules such as MMP-2 and MMP-9 in the placenta (88). Matrix metalloproteinases (MMPs) are proteolytic enzymes associated with a variety of diseases and can promote the production of inflammatory environments.Furthermore, Baczyk et al. (89) found that oxidative stress is associated with SUMOylation. SUMOylation is a reversible post-transcriptional modification of proteins that tags SUMO (a protein consisting of approximately 100 amino acids) to specific peptides by covalent ligation of binding enzymes, thereby regulating protein stability and localization to cells (90, 91). SUMOylation may impair cytoskeleton stability, thereby promoting trophoblast shedding into the maternal circulation (88). SUMOylation may occur when there is oxidative stress at the maternal-fetal interface, such as hypoxia and inflammation. Oxidative stress induced high expression of SUMO-1 and SUMO-4 in the cytoplasm of syncytiotrophoblast cells, while increased expression of SUMO-2 and SUMO-3 in the nucleus.

#### 3.1.5 Correlated signaling pathway

The essential life activities of the human body are composed of many signal pathways. Here, we briefly introduce some signaling pathways related to OS-induced reproductive diseases. Including the p38 MAPK pathway, ASK1-TRX pathway, Jun N-terminal kinase (JNK) pathway, Kelch-like ech-associated protein 1 (Keap1) -nuclear factor erythroid 2-related factor 2 (Nrf2) pathway, Class O family forkhead transcription factors (FOXO), p53 pathway and calmodulin pathway.

Mitogen-activated protein kinase (MAPK) pathway is one of the gene expression regulators in response to oxidative stress. It enables receptor tyrosine kinases, cytokine receptors, and growth factors to interact by controlling the phosphorylation of serine and/or threonine residues (92, 93). The presence of oxidative stress disrupts normal signaling pathways. Studies have found that oxidative stress (OS) induces ROS production and activates the signaling molecule P38 mitogen-activated protein kinase (MAPK), thereby activating the senescence and aging-related inflammation (MMP9) of amniotic mesenchymal cells (AMCs) and chorionic cells (94), resulting in progressive senescence of placental membranes (95). When oxidative stress occurs at the maternal-fetal interface due to various factors, the activation of premature senescence may lead to spontaneous preterm birth and premature rupture of membranes in preterm infants (96, 97). The presence of ROS can also dissociate ASK1-TRX complex by activating kinases (98), but OS-induced p38MAPK activation in amniotic epithelial cells (AECs) is independent of ASK1-TRX signal transconductor, but mediated by TGF-β/TAB1 pathway (62). C-jun N-terminal kinase (JNK) can also be activated by oxidative stress. Hydrogen peroxide (H_2_O_2_) has been found to disrupt the c-jun N-terminal kinase-GST enzyme inhibitory complex, thereby promoting the phosphorylation of JNK (99, 100). When OS occurs, KEAP1-Nrf2 pathway is an important antioxidant defense and protection pathway (101). Nrf2 is activated by the induction of AKT and is a key protective factor against vascular dysfunction and oxidative damage (102). When there is an inflammatory environment of oxidative stress *in utero*, Nrf2 binds more strongly to Keap1 due to its reduced activity, which will weaken the antioxidant system of the body. Deletion of FOXO3 will inactivate Keap1, so some scholars believe that FOXO3 can also affect the KEAP1-NrF2 axis (16). Previous studies have reported that mice lack of p53 also induce preterm birth (103). The p53 pathway is one of the main tumor suppressor pathways regulating cell senescence. The relationship between this pathway and oxidative stress needs to be further demonstrated. Oxidative stress can increase Ca2+ concentration, thereby affecting calmodulin dependent pathways (104).

### 3.2 Adverse effects of oxidative stress on blood vessels

#### 3.2.1 Placental vascular dysplasia

The normal growth and development of placental blood vessels is one of the key factors for healthy pregnancy. The placenta is rich in angiogenic factors and related receptors, such as vascular endothelial growth factor (VEGF), acid/basic fibroblast growth factor (FGF), transforming growth factor (TGF), platelet-derived growth factor (PDGF), placental growth factor (PIGF), tumor necrosis factor (TNF), angloietin, etc. Charnock-jones et al. (105) first detected the expression of VEGF in the endometrium and proposed that VGEF is one of the pro-angiogenic factors secreted by the endometrium. This idea was demonstrated by Zhang et al. (106), who further proposed that decidual natural killer (dNK) cells in direct contact with trophoblast cells could produce a large number of cytokines and pro-angiogenic factors, such as VEGF-A, VEGF-C, interferon-γ (IFN-γ) or placental growth factor (PlGF). These play an important role in protecting embryonic development and the initiation of uterine spiral artery remodeling. When excessive free radicals are produced at the maternal-fetal interface due to various factors, oxidative stress can damage the development of placental blood vessels. Angiogenesis depends on angiogenic factors regulated by estrogen, such as vascular endothelial growth factor (VEGF) (107). The VEGF pathway is closely related to ROS produced by NADP(H) oxidase through REDOX reactions (108). It has been found that when oxidative stress occurs in the placenta, the production of angiogenic factors (placental growth factor, PlGF) and antiangiogenic factors (sflt-1 and sENG) are disturbed, leading to systemic inflammation, endothelial activation, systemic OS and endothelial •NO production changes, respectively (109, 110). In addition, oxidative stress causes the accumulation of advanced oxidation protein products (AOPPs), which affects the normal function of trophoblast cells by promoting trophoblasts to generate soluble FMS-like tyrosine kinase 1 (SFLT-1). This may lead to abnormal development of placental blood vessels and defects in the placental accreta, resulting in placental ischemia, hypoxia and reperfusion injury, and uterine spiral artery remodeling disorder, and then participate in the pathogenesis of preeclampsia (111). During pregnancy, trophoblast cells infiltrate the endometrium, the superficial third of the myometrium, and the associated spiral arteries, and these processes also enable the formation, development, maturation and aging of the placenta. When oxidative stress interferes or blocks trophoblast function during the life cycle of the placenta, pathological pregnancy may occur, such as preeclampsia, fetal growth restriction (FGR) or spontaneous abortion (112, 113). Dysfunction of spiral artery remodeling is an important cause of adverse pregnancy outcomes, such as preeclampsia, fetal growth restriction (FGR), or both. In severe preeclampsia complicated with FGR, only 10% of spiral arteries are completely remodeled, compared with 96% in normal pregnancy.

#### 3.2.2 Immune dysregulation

The maintenance of pregnancy benefits from a balanced state of immune tolerance. Because the decidua contains resident immune cells, decidua cells may maintain the stability of the maternal-fetal interface by providing immune tolerance and can also cause labor by activating inflammatory reactions (103). Killer β-cell immunoglobulin-like receptors (KIRs) on decidual natural killer (dNK) cells are inhibitory receptors that bind specifically to human leukocyte antigen G (HLA-G) on extravillous trophoblast cells in physiological conditions. Inhibiting the killing activity of dNK cells, thereby inducing immune tolerance, plays an important role in protecting embryonic development and the initiation of uterine spiral artery remodeling. Studies have found that when the placenta is in a state of continuous hypoxia, KIRs decreases, the expression level of HLA-G decreases, and the immune activity of dNK cells increases, which leads to excessive apoptosis of trophoblasts through direct killing and exogenous apoptosis pathways, insufficient remodeling of uterine spiral arteries, and blockage of placental circulation.

Oxidative stress can also lead to the activation of various inflammatory transcription factors, thereby causing a vascular immune inflammatory response. When physiological hyperglycemia occurs under conditions such as strenuous exercise, excessive fatigue, high-glucose diet, emotional stress, and alcohol consumption, the level of ROS produced by monocytes increases, leading to increased release of tumor necrosis factor-α (TNF-α) and inflammatory transcription factor nuclear factor-κB (NF-κB). The resulting oxidative stress creates an immune-inflammatory imbalanced environment at the maternal-fetal interface, which may increase the occurrence of insulin resistance (114). Insulin resistance is also one of the important causes of many reproductive endocrine diseases.

#### 3.2.3 Endothelial cell dysfunction

Vascular endothelial cells are a single layer of flat epithelial cells attached to the inner surface of blood vessels and form the inner wall of blood vessels. Endothelial cells are the interface between blood and other vascular walls (tunica media and adventitia) in the lumen of blood vessels. They have a special filtration function and are natural semiselective barriers and circulation factor sensors. In addition, vascular endothelium is also involved in collective immune activities, including vasoconstriction and vasodilation, coagulation (thrombosis and fibrinolysis), angiogenesis, inflammation and swelling, control of leukocyte adhesion and lysis, as well as division and proliferation of other vascular walls (such as tunica media smooth muscle cells) (115). When there is oxidative stress at the maternal-fetal interface, the function of vascular endothelial cells may be impaired.

Previous studies have shown that OS markers O_2_•−, hydrogen peroxide (H_2_O_2_), hydroxyl radical (•OH), nitric oxide (•NO), and peroxynitrite (ONOO−) are involved in the pathophysiology of placental pregnancy diseases. OS at the placental site causes placental injury, and the ischemic placenta releases placental debris, antiangiogenic factors, inflammatory markers, and exosomal vesicles that encapsulate cytotoxic molecules (microRNA proteins) in the maternal circulation. Once in the bloodstream, they meet maternal endothelial cells and release cytotoxic substances that alter cellular expression and lead to inflammation. When endothelial cells are damaged, the permeability of blood vessels increases, and this process is related to the action of TNF-α and thrombin. Ultimately, it may cause endothelial dysfunction and peripheral organ damage throughout the maternal body (116). In the case of preeclampsia, the most severe cases occur in the kidneys, liver, and brain, which can be fatal to the maternal vital organs.

As an important cell signaling molecule, nitric oxide (NO) can affect the relaxation function of blood vessels and make a difference in many life activities. During the second trimester of pregnancy, free radicals are released from the placenta, and when the oxidative and nitrification reactions produced by the placenta are out of balance with the antioxidant system *in vivo*, they affect the release of endothelial nitric oxide (•NO), which may impair placental protection (117) and even induce preeclampsia in the mother (118). Chronic oxidative stimulation will cause cell aging, and when excessive reactive oxygen species are produced in endothelial cells, the expression and function of nitric oxide synthase in endothelial cells will be affected, leading to vasodilation dysfunction (119). Obstruction of vasodilatation can lead to hypertension, insufficiency of perfusion and ischemia of various important organs, and further aggravate oxidative stress. Peroxynitrite (ONOO−) can also change the endothelial expression of some important factors, such as vascular regulatory factors, through posttranslational modification of some endothelial cell proteins, thereby negatively affecting placental vascular function (120).

When oxidative stress occurs, abnormal increases in plasma levels of various endothelial markers can be detected, including cytokines, procoagulants, antiangiogenic factors (SFLT-1 and sENG), and adhesion molecules. The cytokine TNF-α has been shown to cause endothelial cell damage by many studies. Excessive production of ROS plays an important role in causing endothelial dysfunction and tissue damage and regulates the expression of leukocyte adhesion molecules on the surface of endothelial cells (121). Examples include intercellular adhesion molecule-1 (ICAM-1), vascular cell adhesion molecule-1 (VCAM-1), E-selectin (SELE), and P-selectin (SELP). Chen et al. (122) proposed a possible mechanism, that is, with the induction of ROS, endothelial cells regulate the upregulation of ICAM-1, VCAM-1 and chemoattractant protein-1 (McP-1) by secreting TNF-α. It makes white blood cells more prone to adhere to endothelial cells and then produces ROS and various proteolytic enzymes, leading to endothelial cell damage. In addition, Takano et al. (123) also demonstrated that upregulation of intercellular adhesion molecule-1 (ICAM-1) by endothelial cells *via* TNF-α was related to reactive oxygen species (ROS) produced by NADPH oxidase (NOX), while the up-regulation of P-SELP (SELP) is mediated by ROS produced by NOX and xanthine oxidase (XO) commonly. This may be one of the important reasons for microvascular inflammation in physiological conditions.

## 4 Oxidative stress in female reproductive system disorders

### 4.1 Oxidative stress in preeclampsia

Preeclampsia is a common complication of pregnancy, most of which occurs after 20 gestational weeks. Patients may have hypertension and proteinuria, which seriously threatens maternal and infant health. Tsang et al. analyzed placental and hematopoietic cells during pregnancy progression and revealed methods to noninvasively identify pathological changes in placental cells in patients with preeclampsia (55). In the pathogenesis of preeclampsia, the main pathophysiological characteristics are caused by the ischemia induced by poor maternal vascular perfusion (such as decidual artery disease, premature exfoliation and infarction), which is inseparable from placental oxidative stress (124). According to the “six stages of pre-eclampsia” proposed by Redman (125), in the second stage, namely, the formation, development and remodeling of placental blood vessels, we speculate that the abnormality at this stage is probably the core of PE pathogenesis, and the activated source signal molecules can form a cascade amplification effect, making oxidative stress cause continuous damage at the maternal-fetal interface. Therefore, this stage is the golden period for studying the pathophysiological changes of placental blood vessels, and searching for markers of early prediction and diagnosis of PE. Numerous studies have confirmed that arteriolar spasm and vascular endothelial dysfunction are the results of oxidative stress, which is a common pathological manifestation of preeclampsia. Placental hypoperfusion may occur in some patients. Advanced glycation end products (AGEs) interact with receptors (RAGE) in PE and activate NADPH oxidase, leading to the high expression of SFLT-1 in the trophoblast, resulting in increased oxidative stress in PE placenta and forming a vicious cycle (126). Oxidative stress plays a central role in the pathogenesis of preeclampsia. It is not only involved in the first stage of the pathophysiology of preeclampsia, but also related to trophoblast cell invasion and vascular remodeling (127). At the same time, oxidative stress is involved in the second stage of endothelial dysfunction and systemic inflammatory response through the release of proinflammatory factors, cell debris, and other blood circulation (109, 110). When pathological changes occur at the liver, kidney, brain, and placenta levels, clinical manifestations of PE occur (128).

### 4.2 Oxidative stress in intrauterine fetal growth restriction

Preeclampsia is an important cause of intrauterine growth restriction(IUGR), which develops from the mechanism of placental insufficiency and placental ischemia (129). Studies have also shown that placental ischemia/reperfusion injury due to improper development of spiral arterioles can lead to OS in patients with IUGR. The disturbed balance between injury and repair and the abnormal development of villi are the characteristics of IUGR placentas, which are apt to the dysplasia of the syncytial trophoblasts. Syncytiotrophoblast cells are responsible for transport and secretion functions. Therefore, OS is considered an important player in the development of IUGR (130).

### 4.3 Oxidative stress in gestational diabetes mellitus

Gestational diabetes mellitus (GDM) not only has adverse effects on the mother, but also causes more serious secondary diseases such as fetal malformations, premature birth, abnormal growth and development, intrauterine hypoxia, intrauterine fetal death. GDM is closely related to excessive oxidative stress during pregnancy and may lead to placental vascular dysfunction (131). Oxidative stress can mediate insulin resistance (IR) and islet β-cell damage, thereby inducing the occurrence of GDM, and the onset of GDM can lead to higher oxidative stress, promote the development of GDM, and have adverse effects on its intrauterine fetus. Peroxynitrite (ONOO−) is one of the important free radicals produced by nitrification stress. Studies have reported that peroxynitrite (ONOO−) can nitrate proteins and activate MMP-2 and MMP-9 in full-month placentas of type 2 diabetic patients. MMP-9 has been shown to be associated with senescence of chorionic cells (94). When production of ROS is excessive or antioxidant defense is weakened, relatively excessive ROS will damage placental function, such as abnormal angiogenesis and endothelial dysfunction, affect placental perfusion, lead to placental hypoxia, and then result in oxidative stress and GDM during pregnancy (132, 133).

### 4.4 Oxidative stress in spontaneous abortion

Spontaneous abortion is a common adverse pregnancy outcome, with early spontaneous abortion occurring in about 10%-15% of clinical pregnancies (134). Łagód et al. (135) showed in their research that oxidative stress was involved in all stages of spontaneous abortion. By examining the aborted embryo tissue of women, they found that oxidative stress was present. In this process, oxidative stress can affect oocytes, gestational sac implantation, embryo and placental development, placental angiogenesis, and blood flow distribution (136). Qiao et al. (137) found that dysregulation of ALOX15 expression in the placenta will lead to abnormal oxidative stress and placental angiogenesis, which may lead to placental dysfunction and thus contribute to recurrent early pregnancy loss. An experiment on Tet-mev-1 conditional transgenic mice also strongly confirmed that chronic oxidative stress related to abnormal mitochondrial function is one of the important causes of spontaneous abortion and recurrent abortion (138).

### 4.5 Oxidative stress in premature labour

Studies have found that an imbalance of ROS-inflammatory axis can cause preterm birth (139). Inflammation can induce the upregulation of ROS expression and cause significant OS, leading to tissue damage and secondary preterm birth (140). Studies have shown that the concentration of MnSOD compensatively increases as an antagonistic defense response to inflammation and OS, and downregulates NF-Kappa B, activator protein-1 and MAPK pathways (141). Therefore, the detection of Mn-SOD mRNA expression in fetal membranes showed that the expression of fetal membranes in preterm infants was significantly higher than that in full-term spontaneous delivery, which may indicate that a greater degree of OS and inflammation occurs during preterm birth (142). The levels of cytokines IL-1β, IL-6, and IL-8 in the amniotic membrane and chorionic membrane of patients with preterm delivery were significantly higher than those of patients with natural term delivery. These evidences support that preterm birth is associated with activation of the inflammatory response (143).

### 4.6 Oxidative stress in female reproductive system disorders

In addition to its role in common pregnancy complications, oxidative stress may also contribute to other female reproductive disorders. Examples include polycystic ovary syndrome (PCOS) (144), endometriosis (145), and so on. We can assume that the essence of most reproductive diseases is inflammation of the reproductive organs, which makes the homeostasis of proinflammatory and anti-inflammatory factors in the body unbalanced, and in severe cases, promotes cell death. Oxidative stress is one of the important factors leading to inflammation in the body.

## 5 Conclusion

Oxidative stress can act on various stages of the maternal-fetal interface, including hypoxia from early pregnancy, establishment and maturation of maternal-fetal cycle, and participation in proliferation, migration, invasion and apoptosis of trophoblast cells, so that it has become the focus of the current placental pathological pregnancy research. Considering the obvious ethical relationship, oxidative stress in human pregnancy has not been adequately studied. In order to fill the important knowledge gap we lack, more animal model experiments on normal and abnormal pregnancies and the establishment of an *in vitro* culture system for embryos with longer survival time are needed in the future. Moreover, since most of the placental samples obtained in clinic are from the delivery period, and it is quite difficult to obtain samples from other pregnancy periods. In the future, we should find safer and non-invasive detection methods to obtain placental cells and cell products from maternal peripheral blood. With the further development of the study, it is reasonable to speculate that placental genome mutation may be one of the pathogenesis of placental dysfunction, and may also be the key determinant of “major obstetric syndrome” (including pre-eclampsia, fetal growth restriction, stillbirth, etc.). Recently, Sam’s team (146) confirmed that there are unevenly distributed genomic mutations in the placenta, which may be the basis of this genetic aggregation in placental mosaicism. It can be seen from this work that even if the research objects have mosaic phenomenon, different pregnancy outcomes will be observed due to uneven genomic mutations. This also explains why some previous studies have concluded that there is no correlation between placental genome mutations and adverse pregnancy outcomes. In the future, we can further explore the relationship between oxidative stress and placental genome mutation, which may affect villous trophoblasts and further affect blood circulation at the maternal-fetal interface. This also suggests that a more large-scale and systematic study of the genome structure of healthy and diseased human placenta may help to refine the mechanisms of oxidative stress on blood vessels at the maternal-fetal interface, and then determine the role of relevant mechanisms in leading to human placental complications. In addition, we can continue to explore the relationship between the intensity of stressors and the level of oxidative stress during pregnancy, and further improve the quantitative and evaluation methods of mental factors.

Therefore, understanding the relationship between oxidative stress and the mechanism of placental vascular development, exploring the source of oxidative stress at the maternal-fetal interface, and searching for markers reflecting oxidative stress metabolites will help to clarify the pathogenesis of pathological pregnancy and other female reproductive system diseases, and guide the early clinical prediction. Studying the pathways of oxidative stress and the adverse effects of oxidative stress on vascular effects may provide more targeted options for future therapeutic targets. Especially when oxidative stress is considered as an important driver of the development of maternal-fetal interface pathology, antioxidant therapy may be the best treatment for “placental diseases”, and antioxidant lifestyle will help prevent the occurrence of REDOX diseases.

## Author contributions

SY and YZ designed the study. CZ, YG and YY reviewed the literatures and collected the data. CZ, YG and YY wrote and revised the manuscript. CZ, YG, ZD and YF designed the figures. All authors contributed to the article and approved the submitted version.
